# On Capacitance and Energy Storage of Supercapacitor with Dielectric Constant Discontinuity

**DOI:** 10.3390/nano12152534

**Published:** 2022-07-23

**Authors:** Shiqi Zhou

**Affiliations:** School of Physics and Electronics, Central South University, Changsha 410083, China; chixiayzsq@163.com

**Keywords:** ultra-small pore supercapacitor, dielectric discontinuity, differential capacitance, energy storage

## Abstract

The classical density functional theory (CDFT) is applied to investigate influences of electrode dielectric constant on specific differential capacitance $C_{d}$ and specific energy storage E of a cylindrical electrode pore electrical double layer. Throughout all calculations the electrode dielectric constant varies from 5, corresponding to a dielectric electrode, to $\varepsilon_{wr}=$ 10^8^ corresponding to a metal electrode. Main findings are summarized as below. (i): By using a far smaller value of the solution relative dielectric constant $\varepsilon_{r}=10$, which matches with the reality of extremely narrow tube, one discloses that a rather high saturation voltage is needed to attain the saturation energy storage in the ultra-small pore. (ii): Use of a realistic low $\varepsilon_{r}=10$ value brings two obvious effects. First, influence of bulk electrolyte concentration on the $C_{d}$ is rather small except when the electrode potential is around the zero charge potential; influence on the E curve is almost unobservable. Second, there remain the $C_{d}$ and E enhancing effects caused by counter-ion valency rise, but strength of the effects reduces greatly with dropping of the $\varepsilon_{r}$ value; in contrast, the $C_{d}$ and E reducing effects coming from the counter-ion size enhancing remain significant enough for the low $\varepsilon_{r}$ value. (iii) A large value of electrode relative dielectric constant $\varepsilon_{r}^{w}$ always reduces both the capacitance and energy storage; moreover, the effect of the $\varepsilon_{r}^{w}$ value gets eventually unobservable for small enough pore when the $\varepsilon_{r}^{w}$ value is beyond the scope corresponding to dielectric electrode. It is analyzed that the above effects take their rise in the repulsion and attraction on the counter-ions and co-ions caused by the electrode bound charges and a strengthened inter-counter-ion electrostatic repulsion originated in the low $\varepsilon_{r}$ value.

## 1. Introduction

In the vicinity of a charged electrode, electrolyte solution forms an electric double layer (EDL) that reflects the competition between electrostatic attraction of the counter-ions to the electrode surface and the translational entropy of the ions. The EDL is a problem of fundamental importance to subjects as diverse as colloid science [1,2], macromolecular conformation [3,4], and biological membranes [5], and has been a subject of much research interest over the past several decades [6,7,8,9,10,11]. It is well known that charged colloids (i.e., macroions) have typically a low relative dielectric constant ($\varepsilon_{r}$ ≈ 2−5) which is much smaller than that of the surrounding solvent (e.g., for water $\varepsilon_{r}$ ≈ 80). In most of the simulation and theoretical works [12,13,14,15,16,17,18], it is generally assumed that the electrolyte solution and electrode have the same relative dielectric constant.

Usually, the electrode is a metal whose relative dielectric constant is infinite. In this case, the resultant induced charges (caused by the electric field excited by ions in the EDL) on the electrode surface result in an additional electrostatic force on the ions. The electrolyte ions in the EDL excites electric field, which causes a redistribution of the free electrons in the metal; finally, the distribution of the induced charges and salt ions reaches an equilibrium state under the action of the total electric field generated by the induced charges and electrolyte ions. At the same time, the electrostatic potential distribution over the electrode and the EDL region is also determined. If other dielectrics are mixed in the electrode material [19,20] (in order to produce an electrode with a low relative dielectric constant), the bound charge generated by the dielectric due to the polarization of the electric field will also participate in the above process. As for the problem what is the primary electrostatic force on the ions in the solutions, this depends on the ratio of dielectric material to metal material constituting the electrode and the resulting average relative dielectric constant. The lower the electrode relative dielectric constant, the more the bound charges play a major role. From fundamental electrostatics the standard treatment of surface polarization at a dielectric boundary is through imaginary image charges in the medium of the electrode and consequent image forces. Surface polarization forces radically influence the ion distribution and hence, the mean electrostatic potential and surface force [21,22,23,24].

Understanding the surface polarization effect can increase our ability to control the EDL structure and disclose work mechanisms for many practical applications, such as the design of a supercapacitor (SC) [25,26,27,28], modulating three-dimensional conformation of polyelectrolyte brush [29], self-assembly [30,31], ionic profiles [22,23,32], and surface force [21,33,34,35,36,37,38,39].

The present work aims to consider a dielectric discontinuity of the electrode interface in the classical density functional theory (CDFT) framework. The dielectric discontinuity denotes two phases with different dielectric constants in contact; at the phase boundary, the dielectric constant changes discontinuously. The other two quality indicators of the dielectric, dielectric loss and dielectric strength, are not considered, because these characteristics are completely beyond interpretation scope of the CDFT, and they are suitable to be considered by other theories or molecular dynamics simulations [40]. As in the usual electrolyte theories or simulations, we use a general dielectric constant without considering the dependence on temperature and frequency. The CDFT is a convenient starting point for the microscopic structure and thermodynamic properties of inhomogeneous fluids [41,42]. It successfully accounts for the correlation and repulsive volume effects, and is widely applied to many fields of classical statistical mechanics, such as adsorption [43,44,45,46], phase transitions [47,48,49,50,51,52,53,54], inter-surface effective interactions [55,56,57,58], electrical double layer [6,59,60,61,62,63], polymer statistics [64,65,66], and solid [67,68,69].

The dielectric discontinuity CDFT is then applied to investigate influence of the electrode dielectric constant on differential capacitance and energy storage of the EDL inside a cylindrical pore. In the present work, the electrolyte solution is modeled by the primitive model (PM), and the solution relative dielectric constant $\varepsilon_{r}$ is kept fixed at 10.0, this value is far smaller than that of bulk water. This is another novel point of the present work as a considerable part of the works for the SC [16,70,71,72,73] employing aqueous electrolyte solution uses the $\varepsilon_{r}$ value corresponding to that of the bulk water. This is necessary because as the device size becomes smaller and smaller, amount of solvent adsorption near the interface tends to decrease [74]; moreover, correlation of the electric dipole perpendicular to the surface also decreases. It is the two factors [75] that leads to a decrease of the aqueous dielectric constant inside the pore.

Innovation of this work is that the classical density functional theory is applied to dielectric discontinuity for the first time. In an earlier work [76], it was clearly written: “However, the density functional theory and the field theoretic approach have not been applied to the case of the polarized electrode”.

Layout of the paper is structured as follows. The CDFT approach considering the dielectric discontinuity is briefly presented in Section 2; model calculations for influences of the electrode dielectric constant at low bulk dielectric constant value are performed and the relevant results are presented and discussed in Section 3; finally, the main conclusions are summarized in Section 4.

## 2. Model and Method

**Figure 1:**
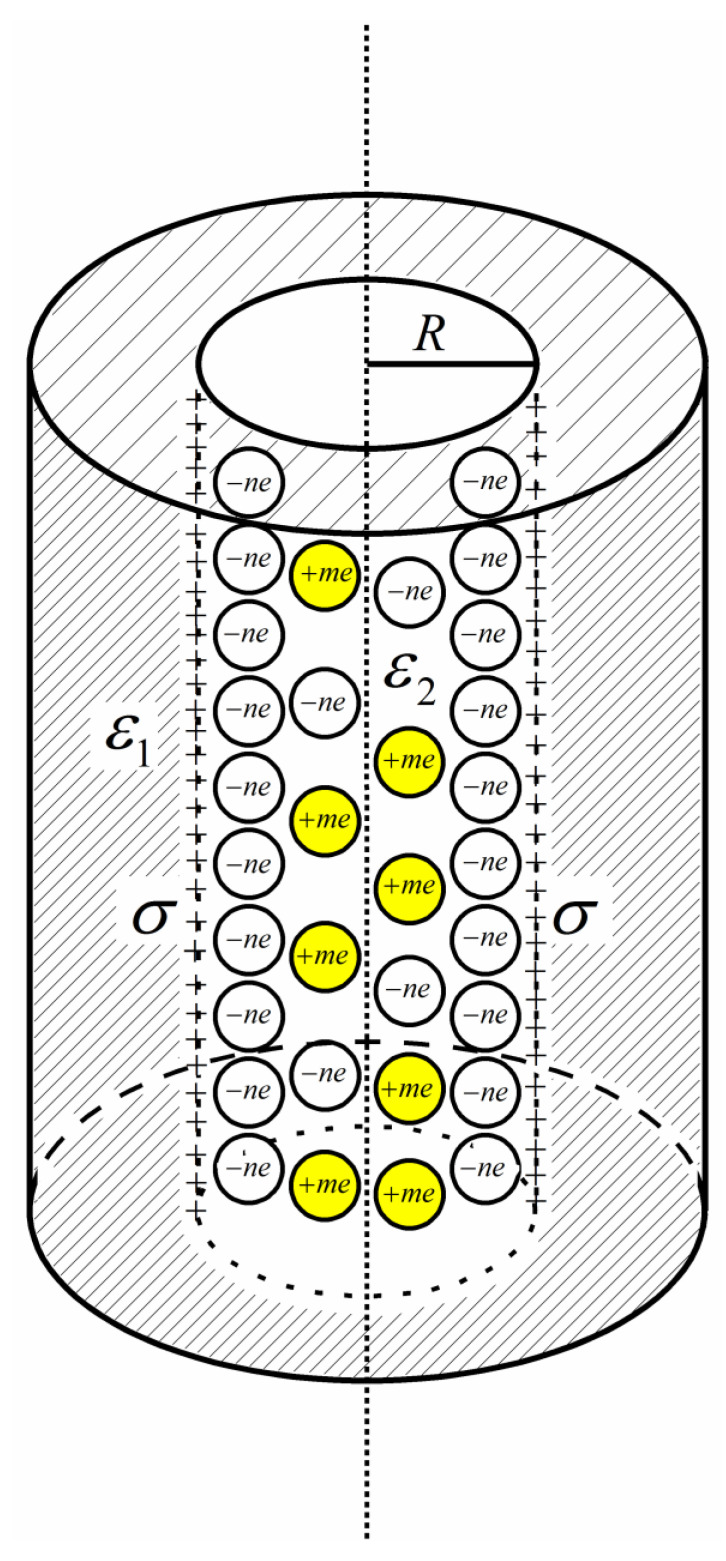
Diagram of the model considered. The electrical double layer is formed by +*m*/−*n* (cation and anion electric valences are, respectively, +*m* and −*n*) PM electrolyte inside an infinitely long cylindrical pore of radius R and with uniformly distributed charges on the inner surface of area charge strength σ. The electrolyte fluid is with medium dielectric constant $\varepsilon_{2}$, and the electrode has dielectric constant $\varepsilon_{1}$.

In the present work, we consider the EDL formed by aqueous electrolyte confined by a cylindrical pore electrode. The pore is infinitely long, and its radius is R. The cylindrical pore wall (i.e., the electrode) is hard and perfectly smooth with dielectric constant $\varepsilon_{1}=\varepsilon_{0}\varepsilon_{r}^{w}$, where $\varepsilon_{0},\;\varepsilon_{r}^{w}$ are vacuum dielectric constant and electrode relative dielectric constant, respectively. The inner is charged with uniform surface charge area density σ. The model is briefly sketched in Figure 1. The aqueous electrolyte is modeled by the so-called PM. In the PM ions are considered as hard spheres (HS) of a diameter $d_{\pm }$. A point electric charge *Z_i_e* is placed at center of the HS. *Z_i_* is the ion charge number (or ion valence) and *e* is the elementary charge strength. The solvent is mimicked by a continuous dielectric medium of dielectric constant $\varepsilon_{2}=\varepsilon_{0}\varepsilon_{r}$ ($\varepsilon_{r}$ is relative dielectric constant contributed by the solvent).

It is usually convenient to describe polarization of the interface by introducing image charges. For spatially confined systems with curved interfaces or for two closely located plane interfaces (for thin interlayers), an accurate description of polarization requires the introduction of an infinite series of image charges. To deal with the dielectric discontinuity for a generic interface, it is necessary to numerically solve the corresponding boundary value problem for the Poisson equation. Although this is usually too time consuming to be feasible in simulations, it does not constitute any computational burden in the CDFT calculations.

The electrostatic potential distribution ψ(r) satisfies the Poisson equation in uniform area $V_{i}$ with dielectric constant $\varepsilon_{i}$ (i=1,2):

(1) $$\nabla^{2}\psi_{i}\left(r\right)=-\frac{\rho \left(r\right)}{\varepsilon_{i}}$$

where ρ(r) is the free charge density in $V_{i}$ which is contributed by mobile electrolyte ions. There are two uniform areas for the present model: $V_{1}$ denotes the electrode, $V_{2}$ denotes the EDL inside the cylindrical electrode pore. At the electrode surface facing the EDL, the electrostatic potential distribution is continuous, i.e.,

(2) $$\psi_{1}\left(r\right)=\psi_{2}\left(r\right)$$

The potential derivative satisfies another boundary value relation:

(3) $$\varepsilon_{2}\frac{\partial \psi_{2}\left(r\right)}{\partial n}-\varepsilon_{1}\frac{\partial \psi_{1}\left(r\right)}{\partial n}=-\sigma$$

$\frac{\partial }{\partial n}$ denotes the partial derivative along normal direction, pointing from $V_{1}$ to $V_{2}$. σ denotes the free charge area density on the interface between $V_{1}$ and $V_{2}$, i.e., the electrode surface charge area density. For metal electrode with $\varepsilon_{r}^{w}=\infty$, because the metal electrode in the state of electrostatic balance is an equipotential body, Equation (3) reduces to:

(4) $$\varepsilon_{2}\frac{\partial \psi_{2}\left(r\right)}{\partial n}=-\sigma$$

By inserting the infinite value of the relative dielectric constant into Equation (1), one infers immediately that within the metal electrode the electric field strength $\vec{E}$ satisfies the following equation:

(5) $$\nabla \cdot \vec{E}=0$$

According to integral transformation, one has:

(6) $$\oint_{S}\vec{E}\cdot d\vec{S}=\int_{V}\nabla \cdot \vec{E}dV=0$$

which, combined with the Gauss theorem, gives that there is no free charge inside the electrode hole. Obviously, this is consistent with the electrostatic equilibrium properties of conductor.

The free charge density ρ(r) is determined by the ion density distribution $\rho_{i}\left(r\right)$ in the EDL:

(7) $$\rho \left(r\right)=\sum_{i=1}^{2}\rho_{i}\left(r\right)Z_{i}e$$

The summation runs over all ion species. The density distribution $\rho_{i}\left(r\right)$ is calculated by minimizing the grand potential $\Omega \left[\left\{\rho_{i}\right\}\right]$. In the CDFT, the $\Omega \left[\left\{\rho_{i}\right\}\right]$ is supposed to be a functional of $\rho_{i}\left(r\right)$, and is related to the intrinsic Helmholtz free energy $F\left[\left\{\rho_{i}\right\}\right]$ via the Legendre transform

(8) $$\Omega \left[\left\{\rho_{i}\right\}\right]=F\left[\left\{\rho_{i}\right\}\right]+\sum_{i}\int dr\rho_{i}\left(r\right)\left(u_{wi}\left(r\right)-\mu_{i}\right)$$

where $\mu_{i}$ is chemical potential for the i type ion, and $u_{wi}\left(r\right)$ is an external potential acting on the i type species, and for the homogeneously charged and hard cylindrical pore surface of the present consideration, it is calculated as follows:

(9) $$u_{\mathrm{wi}}\left(r\right)=\{\begin{array}{ll} \infty , & r>R-d_{i}/2\\ Z_{i}e\psi_{w}, & r<R-d_{i}/2 \end{array}$$

where $\psi_{w}$ is the electrostatic potential generated by the electrode surface charge area density σ, and does not depend on *r* because of the columnar symmetry of the electrode charge distribution. The value of $\psi_{w}$ is not trivial because it influences the density distribution $\rho_{i}\left(r\right)$ in the EDL; the value can be determined by the electrical neutral condition.

The intrinsic Helmholtz energy $F\left[\left\{\rho_{i}\right\}\right]$ includes the ideal gas contribution and the excess contribution $F_{ex}$. The former is analytically available from textbook of statistical thermodynamics, and the latter originates from internal interactions within the system, and its acquirement has to resort to approximations. In the present calculations, we use the density functional approximations tested in literatures [77,78,79,80]. In detail, the hard sphere repulsion coming from the internal inter-ion short-range interaction is treated by a well confirmed fundamental measure functional, the long-range inter-ion electrostatic interaction is dealt with by mean field approximation, and the interplay between the hard sphere repulsion and electrostatic interaction is calculated by a second order functional perturbation expansion whose expansion coefficient is exactly the bulk second order direct correlation function based on a mean spherical approximation closure to the Ornstein-Zernike integral equation. Relevant details are recorded in literatures [77,78,79,80] and not repeated here. Particularly, literature [80] indicates that the electrical capacitance properties of extreme nanoscale SC can still be predicted rather reliably even the prediction of density distribution becomes worse under the extreme condition.

After numerical solution of the Poisson equation and minimization of the grand potential $\Omega \left[\left\{\rho_{i}\right\}\right]$, both the density distribution and electrostatic potential distribution profiles in equilibrium are available. The differential capacitance per unit area $C_{d}$ is calculated according to definition:

(10) $$C_{d}=\frac{\partial \sigma }{\partial \psi_{2}\left(R\right)}$$

where, $\psi_{2}\left(R\right)$ is the electrode potential, which is defined with reference to potential of a bulk electrolyte.

Accordingly, the energy stored per unit area is calculated as:

(11) $$E\left(U\right)=\int_{0}^{U}U_{s}C_{d}\left(U_{s}\right)dU_{s}$$

where the U is the final electrode potential, whereas the above $\psi_{2}\left(R\right)$ is only used to calculate the $C_{d}$ value. So, the $\psi_{2}\left(R\right)$ value can be any value between zero and U.

## 3. Results and Discussion

**Figure 2:**
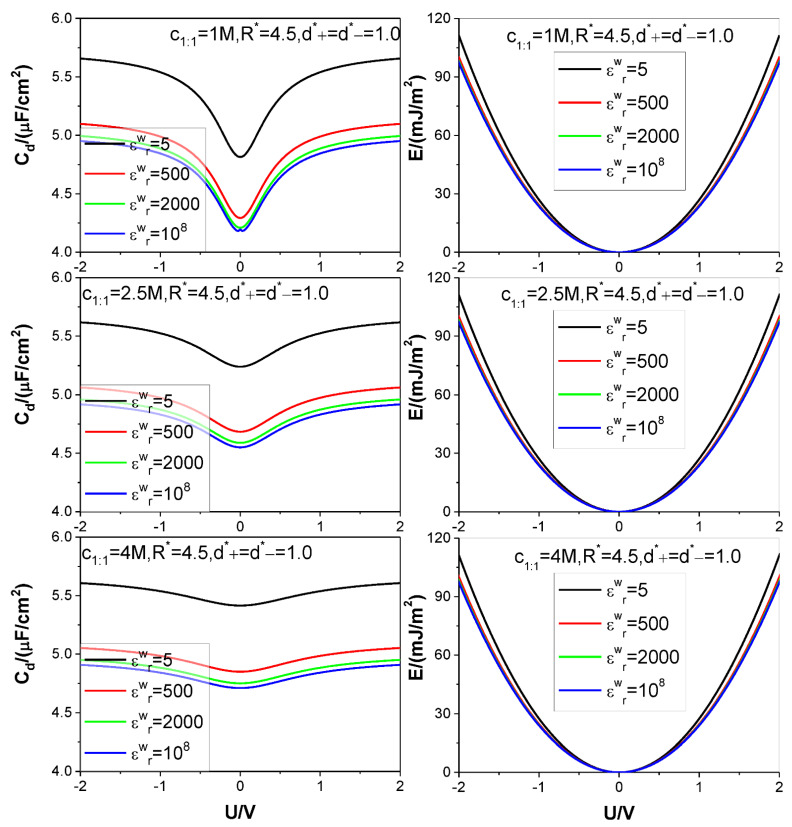
Curves of specific differential capacitance $C_{d}$ and specific energy storage density E. Three values of bulk +1:−1 salt mole concentration and four values of the electrode relative dielectric constant $\varepsilon_{r}^{w}$ are considered, as marked in the figures. Values of other parameters are marked in the text and figures, respectively.

**Figure 3:**
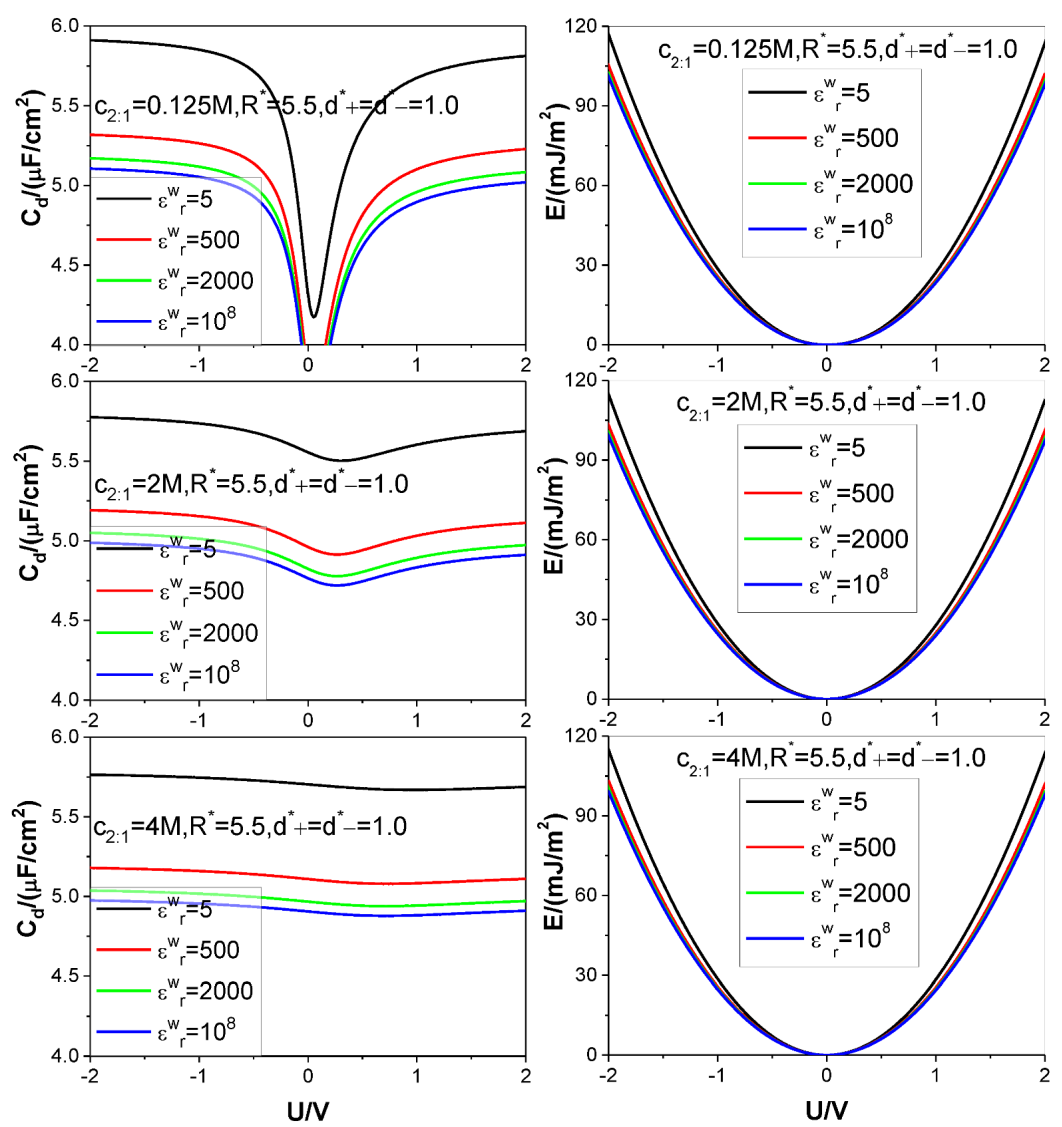
Same as in Figure 2 except that +2:−1 salt is considered. Values of other parameters are marked in the text and figures, respectively.

**Figure 4:**
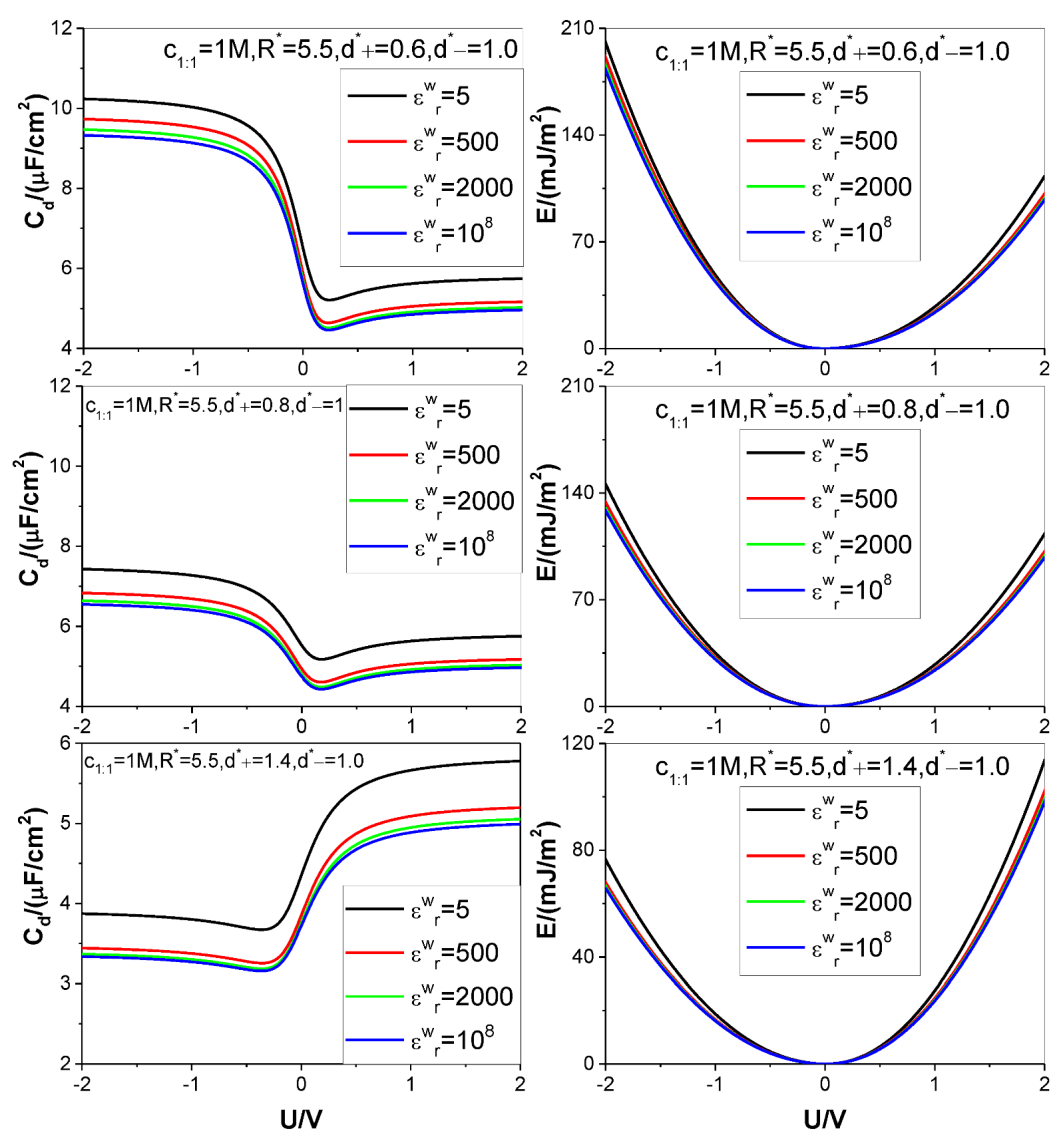
Same as in Figure 2 except that three values of diameter of cation (+1:−1 salt) are considered. Values of other parameters are marked in the text and figures, respectively.

**Figure 5:**
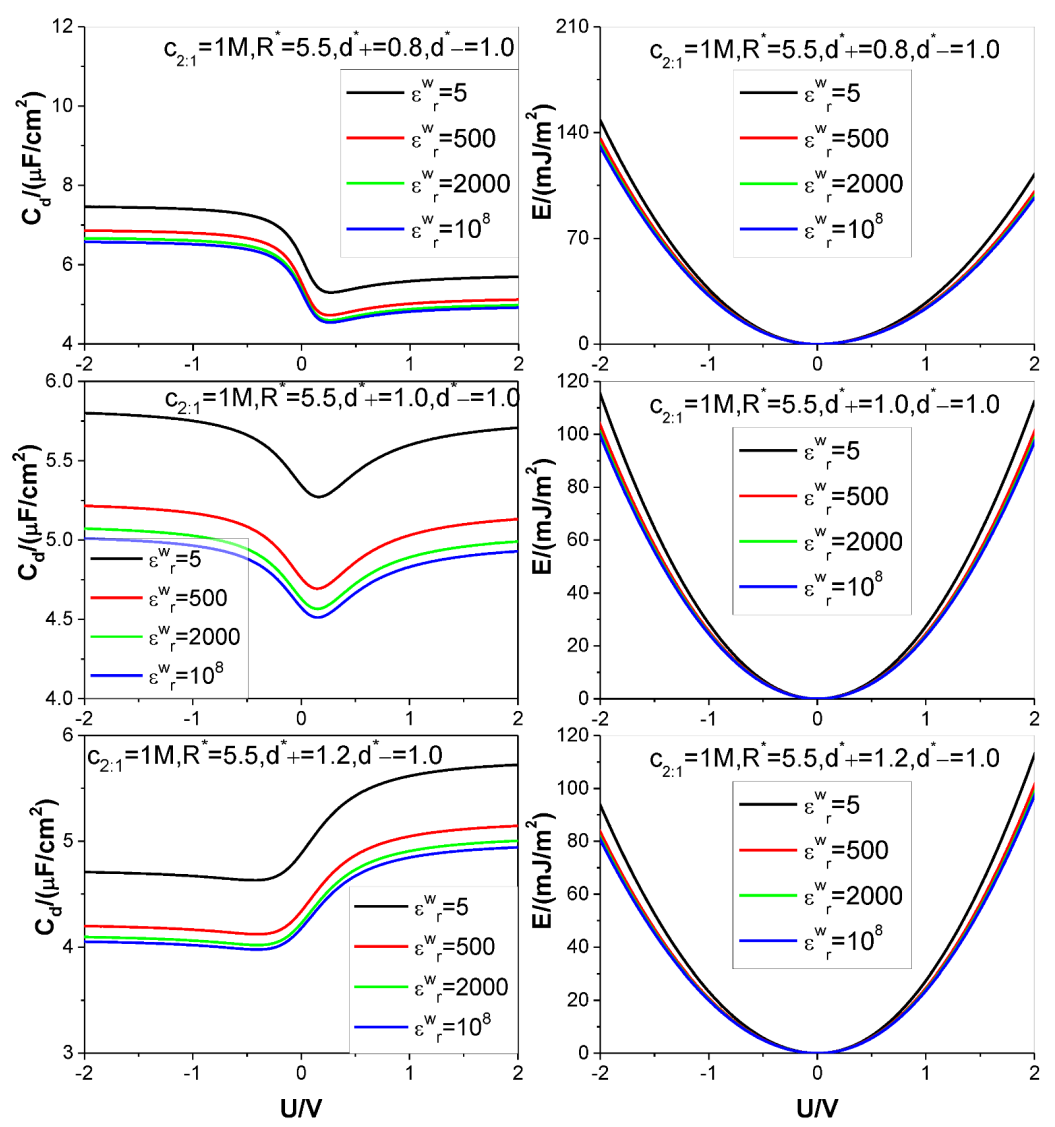
Same as in Figure 2 except that three values of diameter of cation (+2:−1 salt) are considered. Values of other parameters are marked in the text and figures, respectively.

**Figure 6:**
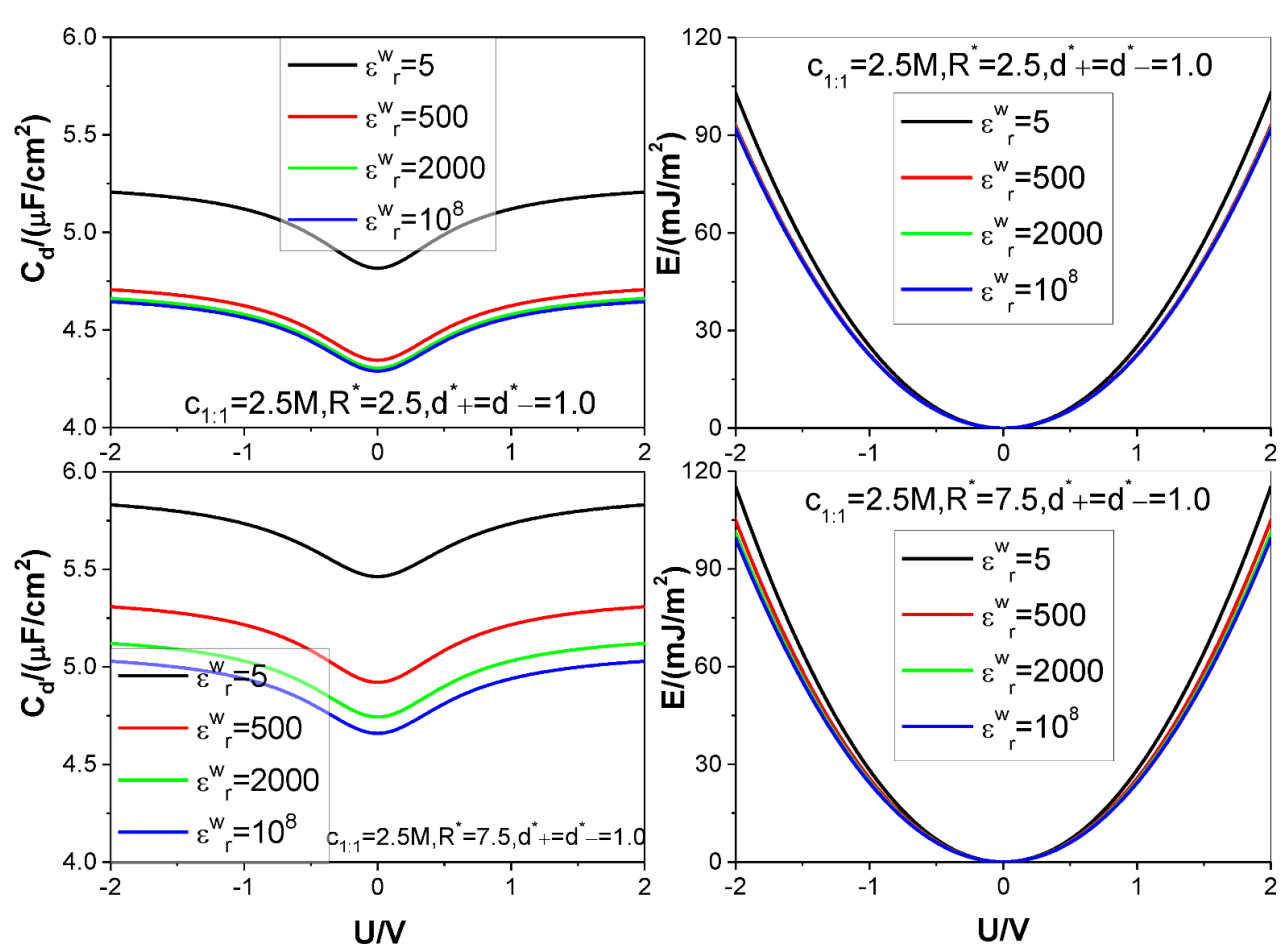
Same as in Figure 2 except that two values of reduced pore radius $R^{*}$ are considered. Values of other parameters are marked in the text and figures, respectively.

Four different values of the electrode relative dielectric constant $\varepsilon_{r}^{w}$ are used, namely, $\varepsilon_{r}^{w}=$ 5, 500, 2000, and 10^8^, respectively, among which, $\varepsilon_{r}^{w}=$ 10^8^ corresponds to a metal electrode, $\varepsilon_{r}^{w}=$ 5 denotes a dielectric electrode. The ion diameters considered are around $d=4\times 10^{-10}\;m$, so we use d as the length unit to non-dimensionalize the cation and anion diameters $d_{+}$ and $d_{-}$, respectively: the relevant reduced diameters are $d_{+}^{*}=d_{+}/d$ and $d_{-}^{*}=d_{-}/d$; the electrode surface charge area density σ is reduced as $\sigma^{*}=\sigma d^{2}/e$ (e is elementary charge strength); both counter-ion and co-ion adsorptions $\Gamma_{counter-ion}$ and $\Gamma_{co-ion}$ are reduced as $\Gamma_{counter-ion}^{*}=\Gamma_{counter-ion}^{}d^{2}$ and $\Gamma_{co-ion}^{*}=\Gamma_{co-ion}^{}d^{2}$, respectively. Four values are considered for the reduced pore radius $R^{*}=R/d$: 2.5, 4.5, 5.5, and 7.5. +*m*:−*n* type electrolyte is considered, several representative values are chosen for the relevant bulk mole concentration $c_{m:n}$: *125 M,1 M,2,5 M,4 M*. The thermodynamic temperature and electrolyte relative dielectric constant $\varepsilon_{r}$ are fixed at 298.15 K and 10, respectively. For clarity and comparison, we summarize the parameter combinations corresponding to each of Figure 2, Figure 3, Figure 4, Figure 5 and Figure 6 in Table 1.

**Figure 7:**
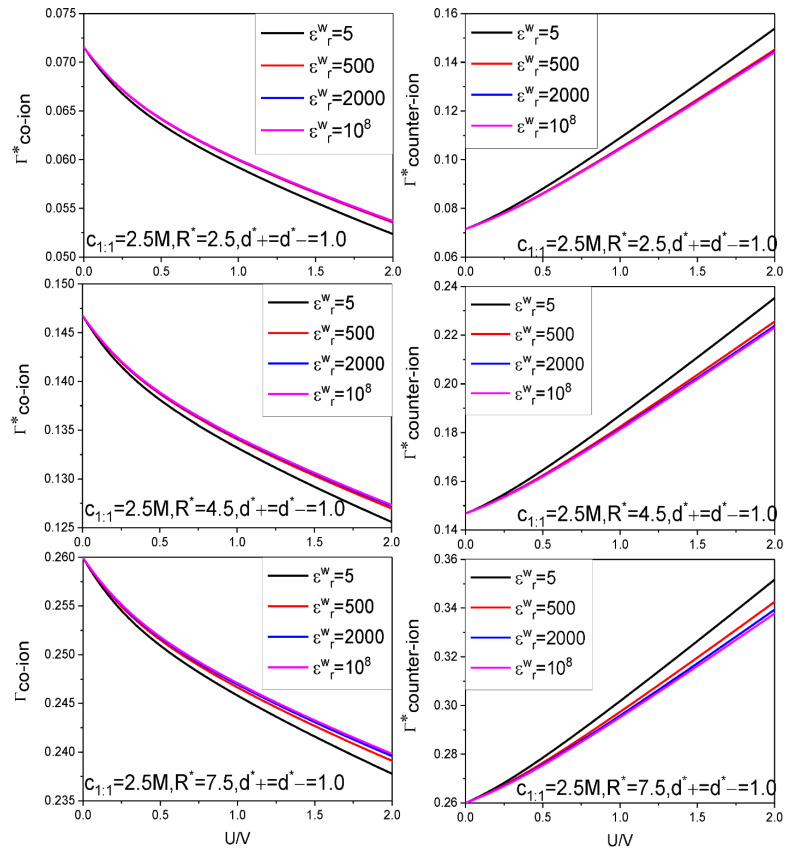
Co-ion and counter-ion adsorption capacities as a function of electrode surface potential. Three cylindrical pore radii and four values of the electrode surface relative dielectric constant are considered. Values of other parameters are marked in the figure.

**Figure 8:**
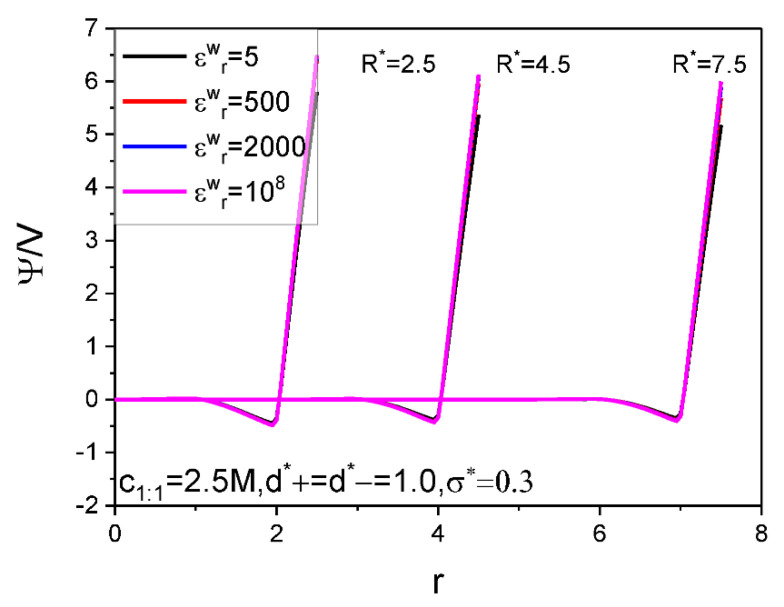
Electrostatic potential profiles within the pore with three different pore radii. The reduced electrode surface charge density is fixed as $\sigma^{*}=0.3$, and four values of the electrode surface relative dielectric constant are considered. Values of other system parameters are marked in the figure.

The calculation results for the SC specific differential capacitance $C_{d}$ and specific energy storage E are presented in Figure 2, Figure 3, Figure 4, Figure 5 and Figure 6. We will summarize the outcomes caused by the low solution $\varepsilon_{r}^{}$ value and different values of the electrode relative dielectric constant $\varepsilon_{r}^{w}$ by giving a detailed analysis on the present results and comparing the present results with those based on bulk aqueous $\varepsilon_{r}$ value, as published previously. To support the analysis, we present in Figure 7 and Figure 8 the co- and counter-ion adsorption curves and electrostatic potential profiles within the pore for several parameter combinations in Table 1.

One of the most significant changes caused by the low $\varepsilon_{r}$ value is that both the E value and the $C_{d}$ value reduce greatly with the low $\varepsilon_{r}$ value dropping. Generally speaking, previous studies indicate repeatedly [73,74] that the $C_{d}$ value falls within order of magnitude of $F/m^{2}$ for the normal aqueous $\varepsilon_{r}$ value, such as $\varepsilon_{r}=78.5$, whereas for the low $\varepsilon_{r}$ value, as considered presently, such as $\varepsilon_{r}=10$, the $C_{d}$ value falls within order of magnitude of $\mu F/cm^{2}$. i.e., the $C_{d}$ value goes down two orders of magnitude for approximately equal electrode potential. Corresponding to this, the E value at voltage of 2 V reduces to one twentieth of the E value corresponding to $\varepsilon_{r}=78.5$ [73]. We will explore the relevant action mechanisms by analyzing the ion adsorption inside the pore and the space electrical potential distribution. The principle of electrostatic field points out that strength of the electrical potential generated by a point charge is in inverse proportion to the medium $\varepsilon_{r}$ value. Given equal ion distribution profile and surface charge distribution the electrical potential strength will increase with dropping of the medium $\varepsilon_{r}$ value. As a result, the curve of the voltage versus surface charge becomes less steep; the $C_{d}$ value and E value reduce accordingly. On the other hand, with decreasing of the solution medium $\varepsilon_{r}$ value, inter-ionic electrostatic interaction strength increases. Considering that the counter-ions dominate the pore unless around a zero charge potential (ZCP), on the average, the inter-counter-ion electrostatic interaction gets more and more repulsive with decrease of the $\varepsilon_{r}$ value. As a result, the inter-counter-ion average separation tends to enlarge to lower the system energy; moreover, the effect increases with decrease of the $\varepsilon_{r}$ value. Consequently, the counter-ion adsorption capacity is lower in $\varepsilon_{r}=10.0$ than in $\varepsilon_{r}=78.5$, and from the point of ion-filling, both the $C_{d}$ and E values necessarily reduce with the decrease of the $\varepsilon_{r}$ value, as it is.

It is noted that the bulk mole concentration influence is far smaller in $\varepsilon_{r}=10.0$ than in $\varepsilon_{r}=78.5$. The weakening of the influence is reflected in two aspects. First, compared with the $U-C_{d}$ curves for $\varepsilon_{r}=78.5$, which change from the camel-shaped to bell-shaped with the bulk concentration changing from 1 M to 4 M, it is difficult for the $U-C_{d}$ curves with $\varepsilon_{r}=10.0$ to finish the shape transition over the same concentration range. However, the trend of the $U-C_{d}$ curve shape transition is the same, i.e., with the bulk concentration increasing, the well depth of the camel-shaped curve around the ZCP gets shallower and shallower, and the shape transition trend will eventually emerge. The reason why concentration induces the transition from camel shaped curve to bell shaped curve near ZCP is that concentration is an adsorption driving force. Even if there is no electric field potential energy, enough ions can be adsorbed near the electrode by bulk concentration alone, so that a capacitance peak appears near the ZCP. Now, due to the relatively low dielectric constant value used in the calculation, it undoubtedly increases the Coulomb repulsion energy between the adsorbed ions, thus reducing the amount of ion adsorption near the ZCP, making it difficult for the peak capacitance to appear. Second, influence of the bulk concentration on the U−E curve is almost unobserved. This is not in contradiction with the $U-C_{d}$ curve concentration dependence. The $U-C_{d}$ curve change caused by the bulk concentration is mainly around the ZCP, as shown in the Figure 2 and Figure 3; as a result, the $C_{d}$ change with the bulk concentration does not cause obvious change of the integrand function in the integral in Equation (9). So, the influence of the $C_{d}-U$ curve change does not cause obvious change of the U−E curve, as it is. Both the bulk concentration and the voltage serve as driving force for the ion adsorption. Because the low $\varepsilon_{r}$ value strengths the electrostatic interaction between the surface charge and the ion, the bulk concentration influence is necessarily weakened. It is observed from Figure 3 that the $C_{d}-U$ curve is no longer symmetrical around the zero potential point, this is due to the ion electric valence asymmetry. However, the minimum of the $C_{d}$ does not occur at U = 0 V. From the Figure 3, the minimum potential moves to certain positive potential without exception, this is obviously related to the electric valence difference. When the bulk mole concentration is very low, the adsorption capacity is very low at the zero charge potential because the driving force coming from the concentration difference is very low. As a result of the very low adsorption capacity, the electric valence asymmetry effect cannot be displayed, the $C_{d}$ minimum still occurs at U = 0 V. With increasing of the bulk mole concentration, the driving force from the concentration difference increases; correspondingly, the adsorption capacity at zero charge potential is no longer negligible, and well depth of the $C_{d}-U$ curve reduces [81]. At very close to the zero charge potential, also because of the non-negligibility of the adsorption capacity, the electric valence asymmetry effect is displayed more and more obviously. Because the anion charge strength is lower than the cation one, at positive potential electrode, whose counter-ion is anion, the relevant $C_{d}$ value is necessarily lower than that at negative potential electrode. The final outcome is that the $C_{d}$ minimum occurs at an appropriate positive potential electrode.

For asymmetrical electrolytes, such as those with ion valency or ion size asymmetries, both the $U-C_{d}$ and U−E curves become asymmetrical w.r.t. the ZCP, as expected. In detail, higher ion valency and/or smaller ion size help(s) in raising the $C_{d}$ and E values whether the bulk relative dielectric constant $\varepsilon_{r}=10$ or $\varepsilon_{r}=78.5$. The main difference is that the counter-ion valency effect in $\varepsilon_{r}=10$ becomes far less obvious than when it is in $\varepsilon_{r}=78.5$. As a result, the curve asymmetry caused by the valency asymmetry is not so obvious, as when in $\varepsilon_{r}=78.5$. The difference originates from a balance between two factors working in opposite directions. On one hand, the counter-ion of higher valency will increase the charge storage given the same ion adsorption capacity. On the other hand, high electrical valency helps in raising the inter-counter-ion electrostatic repulsion, and this tends to decrease the ion adsorption capacity and accordingly the charge storage; moreover, the trend is reinforced by the low bulk $\varepsilon_{r}$ value. As a result, of the two factors, the one restraining the increasing of the charge storage can show its effect better in $\varepsilon_{r}=10$ than in $\varepsilon_{r}=78.5$. However, one may ask: why the counter-ion size effect remains significant enough in $\varepsilon_{r}=10$? It is well-known that the hard sphere repulsion is an interaction far stronger than the electrostatic interaction; as a result, change of the latter strength caused by the lowering of the $\varepsilon_{r}$ value, is far weaker than that caused by the former.

For aqueous component electrolyte solution, the electrolysis will occur at the electrode once a voltage of approximately 1.25 V is applied. However, recent effort succeeds in expanding the electrochemical window of aqueous electrolytes [82]. Moreover, by increasing hydrophobicity of the ion component by using ionic liquid or molten salt, the electrochemical window can become wider. Molten salt and some of the ionic liquids also can be modelled by the PM. Although these nonaqueous electrolyte systems have lower relative dielectric constant, the present calculations are performed with low relative dielectric constant. So, considering the limitations caused by the convergence of algorithm used, the present calculations are performed over a range of the voltage from −2.0 V to 2.0 V. From Figure 2, Figure 3, Figure 4, Figure 5 and Figure 6, one knows that at the maximum voltage considered the $C_{d}$ value is far from approaching zero, and the E value is far from saturation, and actually is still on the rise. As a result, it is confirmed that a low $\varepsilon_{r}$ value significantly increases the saturation voltage, beyond which the E value does not further increase with the voltage applied. The essential reason for the above phenomenon is that the low $\varepsilon_{r}$ value strengths the inter-ionic electrostatic interaction, and accordingly the electrostatic repulsion between the counter-ions, the dominating ions in the pore, increases. Consequently, to keep the counter-ions accommodated in the pore in an energetically favorable way, a higher voltage is needed to offset the electrostatic repulsion between the counter-ions by the electrostatic attraction between the surface charge and the counter-ions. Although a gentle U−σ curve always enables a small value of the $C_{d}$, it causes a higher value of the voltage corresponding to the pore closely packed state, as analyzed above. It is known that the energy storage is roughly proportional to square of the voltage, but only proportional to the $C_{d}$ value; so, the high value of the saturation voltage caused by the low $\varepsilon_{r}$ value significantly increases the saturation E value. However, to make high voltage value practically possible, one must use suitable nonaqueous electrolyte with a wider voltage window. Usually, hydrophobic ionic liquids are of a stable electrochemical window up to 6 V. In fact, the ionic liquids are usually free of polar solvent, value of the relevant $\varepsilon_{r}$ is probably even lower than the present $\varepsilon_{r}=10$ to be responsible for accounting for the electronic polarization of the ions.

It is found that with the decreasing of the electrode $\varepsilon_{r}^{w}$ value, both the $C_{d}$ and E values rise monotonously for all voltages considered. Generally speaking, for voltage strength of 2 V, an E value increase rate of 15% can be achieved by reducing the $\varepsilon_{r}^{w}$ value from $10^{8}$ corresponding to a metal electrode to 5 corresponding to a dielectric electrode. It should be pointed out that the electrode surface is covered with electrolyte, the electrode material largely affects the transfer of charge. So, the 15% increase rate is obtained at the cost of slowed dynamic process. However, two points analyzed below make one believe that decreasing the electrode $\varepsilon_{r}^{w}$ value is still an effective way to improve synthetic performance of the SC. First, obviously, from the changing trend of the U−E curves, one can expect that the E increase rate will rise even faster with the voltage. Considering that a high saturation voltage value is always associated with the low $\varepsilon_{r}$ value electrolyte, and the relevant stable electrochemical window for some ionic liquids can be up to 6 V, as analyzed above, the E value increase rate far higher than 15% can be achieved. Second, exactly, use of the dielectric electrode may slow down the relevant dynamic process; so it is necessary to strike a balance between the energy storage density and power density of the SC. Fortunately, use of the SC is partially encouraged by its exceptionally high power density and fast charging and low effective series resistance, so there is room to move the balance point to be favorable for the E value increase rate. Further work is needed to determine the optimal parameter combination to solve this dilemma. As for why a low electrode $\varepsilon_{r}^{w}$ value always causes both the $C_{d}$ and E to rise, it can be analyzed from the perspective of dielectric polarization. With a given σ value, the higher the $\varepsilon_{r}^{w}$ value is, the more intense the electric polarization vector P caused. According to formula: $\left|\sigma^{\prime }\right|=\left|P_{n}\right|$ ($P_{n}$ is the normal component of P), the bound charge area density strength $\left|\sigma^{\prime }\right|$ rises with the $\varepsilon_{r}^{w}$ value. Consequence of this correlation is that more co-ions can be accumulated near the electrode surface, and at the same time, the counter-ion adsorption in the vicinity of the electrode drops somewhat because the counter-ions are repelled by the likely charged bound charges when they approach the interface, and the presence of the co-ion at the electrode surface leaves less space for the counter-ion to stay there. The two changes work in the same direction and help in raising the surface electric potential strength. On the other hand, the sign of the bound charge is always opposite to that of the electrode surface charge, so presence of the bound charge always tends to reduce the surface electric potential strength. However, the double effects occurring inside of the pore overpasses the single effect occurring outside of the pore, so the net effect is that the surface electric potential strength increases with the $\varepsilon_{r}^{w}$ value under circumstance of fixed σ value, and accordingly, both the $C_{d}$ and E values drop with the $\varepsilon_{r}^{w}$ value, as it is. Figure 7 clearly shows the positive correlation between the co-ion adsorption and the $\varepsilon_{r}^{w}$ value and the negative correlation between the counter-ion adsorption and $\varepsilon_{r}^{w}$ value.

Figure 6 displays the pore size effect on the differential capacitance and energy storage. It is shown that the $U-C_{d}$ curves move up as a whole with the R value. It is known that a larger pore is always associated with a smaller curvature, which makes for easy gathering of the counter-ion around the electrode surface; this necessarily contributes to reduce the surface electrical potential strength, as clearly shown in Figure 8, and increase the electrical capacitance, as it is. The E value is positively correlated with the pore size. This is expected because the larger the pore size, the more the ions can be accommodated; with increasing of the voltage, more and more co-ions are excluded out of the near electrode region, instead, more counter-ions are adsorbed, as shown in Figure 7. Consequently, inter-counter-ion electrostatic repulsions play an increasingly important role; moreover, the repulsions are strengthened by the low $\varepsilon_{r}$ value. As a result, the pore size effect is not as big as expected as the enhanced repulsions between the counter-ions enlarge the inter-counter-ion separation and accordingly reduce the pore space utilization and the counter-ion adsorption capacity. With further increase of the voltage applied, the electrostatic attraction between electrode surface and the counter-ions increases and this helps in offsetting partially the inter-counter-ion electrostatic repulsions and making the inter-counter-ion separation reduce and the counter-ion adsorption increase. Consequently, the pore size effect on the E value gets more and more significant with the voltage for low $\varepsilon_{r}$ value. It is also shown that the strength of the $\varepsilon_{r}^{w}$ value effect is closely related with the pore size; particularly, the $\varepsilon_{r}^{w}$ value effect gets eventually unobservable for small enough pore if the $\varepsilon_{r}^{w}$ value differs from that corresponding to the dielectric electrode. This is expected because the electrostatic attraction between the electrode surface and the counter-ions is so strong in small size pore that the counter-ions can be closely packed regardless of the $\varepsilon_{r}^{w}$ value. As a result, even if the $\varepsilon_{r}^{w}$ value changes very much, the counter-ion absorption does not change to the same extent, and so do both the $C_{d}$ and E, as it is.

We have not found literatures that provide the correlation of $C_{d}-U$ and E−U curves and electrode dielectric constant. Usually, the electrode material is made of metal. With the progress of electrode manufacturing technology, electrodes mixed with non-metallic materials and with adjustable dielectric constant will become more and more common. Our data show the negative correlation between the electrode dielectric constant and the energy storage of the SC and other concomitant effects. So, the electrode dielectric discontinuity certainly elicits new changes of multiple dilemmas such as energy-power-size-hysteresis; [83] solving these dilemmas will be new growth point of the SC field.

## 4. Summary

The present treatment about the dielectric discontinuity is rather general and applies for any version of the CDFT as we account for the dielectric discontinuity issue by solving numerically the boundary value problem of the Poisson equation. We employ the PM for the electrolyte solution with a relative dielectric constant $\varepsilon_{r}$ being fixed at 10.0, far smaller than that of the bulk water, to reflect the actual situation of extremely small size pore. Consequently, the present results are of practical significance. The main conclusions are summarized as follows.

(i) The low solution $\varepsilon_{r}$ value greatly reduces both the $C_{d}$ value and the E value under certain electrode potential, but at the same time, significantly increases the saturation voltage, beyond which the E value does not further increase with the voltage applied, which significantly increases the saturation E value because of the approximate proportional relation between the energy storage and square of the voltage in comparison to the proportional relation between the energy storage and the $C_{d}$ value.
(ii) Because of the low solution $\varepsilon_{r}$ value, influence of electrolyte bulk concentration on $C_{d}$ is rather small except when the electrode potential is around the ZCP; consequently, the energy storage curves are rather insensitive to the electrolyte bulk concentration.
(iii) Higher counter-ion valency or smaller counter-ion size help in raising the $C_{d}$ and E values. The enhancing effect of the counter-ion valency reduces greatly with dropping of the $\varepsilon_{r}$ value; whereas the counter-ion size effect remains significant enough for low $\varepsilon_{r}$ value.
(iv) Both the $C_{d}$ and E values increase monotonously with the electrode dielectric constant $\varepsilon_{r}^{w}$ decreasing for all voltages considered; the E increase rate with the dropping of the $\varepsilon_{r}^{w}$ value rises faster with the voltages, and for voltage strength of 2 V the E value increase rate up to 15% can be achieved by reducing the $\varepsilon_{r}^{w}$ value from $10^{8}$ to 5. For small enough pore the $\varepsilon_{r}^{w}$ value effect gets unobservable when the $\varepsilon_{r}^{w}$ value differs from that corresponding to dielectric electrode.
(v) Both the $C_{d}$ and the E values are positively correlated with the pore size; but for the low $\varepsilon_{r}$ value considered the pore size effect on the E value gets more and more significant with the voltage applied.

## Figures and Tables

**Table 1:** Summary of studied parameters.

Figure No	c/mol L^−1^	$\varepsilon_{r}^{w}$	R/d	$d^{+}/d$	$d^{-}/d$	m:n	T/K	$\varepsilon_{r}^{}$
Figure 2	1.0	5, 500, 2000, 10^8^	4.5	1	1	1:1	298.15	10
	2.5							
	4.0							
Figure 3	0.125	5, 500, 2000, 10^8^	5.5	1	1	2:1	298.15	10
	2.0							
	4.0							
Figure 4	1.0	5, 500, 2000, 10^8^	5.5	0.6	1	1:1	298.15	10
				0.8				
				1.4				
Figure 5	1.0	5, 500, 2000, 10^8^	5.5	0.8	1	2:1	298.15	10
				1.0				
				1.2				
Figure 6	2.5	5, 500, 2000, 10^8^	2.5 7.5	1	1	1:1	298.15	10

## Data Availability

The data that support the findings of this study are available from the corresponding author upon reasonable request.
